# Endoscopic ultrasound in the diagnosis of cholangiocarcinoma as the etiology of biliary strictures: a systematic review and meta-analysis

**DOI:** 10.1093/gastro/gou057

**Published:** 2014-08-27

**Authors:** Udayakumar Navaneethan, Basile Njei, Preethi GK Venkatesh, Vennisvasanth Lourdusamy, Madhusudhan R Sanaka

**Affiliations:** ^1^Digestive Disease Institute, The Cleveland Clinic, Cleveland, OH, USA and; ^2^Department of Internal Medicine, University of Connecticut Health Center, Farmington, CT, USA

**Keywords:** endoscopic ultrasound, fine-needle aspiration, cholangiocarcinoma

## Abstract

**Background and aim:** Extrahepatic cholangiocarcinoma (CCA) typically presents as biliary strictures. Endoscopic ultrasound (EUS)-fine needle aspiration (FNA) may contribute to the diagnosis of CCA as the etiology of extrahepatic biliary strictures. Our aim was to study the uselfulness of EUS-FNA in diagnosing CCA as the etiology of biliary strictures.

**Patients and methods:** In this meta-analysis, PUBMED and EMBASE databases were examined to find studies published to April 2014 where diagnostic correlation of CCA was available. Studies reporting only “positive for malignancy” were included in our analysis. The main outcome measurements were sensitivity, specificity and likelihood ratio.

**Results:** Six studies were included, covering 196 patients. The overall pooled sensitivity and negative likelihood ratio (LR-) of EUS-FNA for diagnosis of CCA were 66% [95% confidence interval (CI) 57–74%] and 0.34 (95% CI 0.26–0.43), respectively. In five studies (146 patients), where a mass lesion was detected during EUS, the pooled sensitivity and LR- of EUS-FNA for diagnosis of CCA were 80% [95% CI 72–87%] and 0.20 (95% CI 0.13–0.28), respectively. In the 49 patients with a negative brush cytology, the pooled sensitivity and LR- of EUS-FNA for diagnosis of CCA were 59% [95% CI 44–73%] and 0.41 (95% CI 0.27–0.56), respectively.

**Conclusions:** Our study suggests that EUS-FNA is useful in the evaluation of CCA as the etiology of biliary strictures. EUS-FNA may improve the diagnosis of CCA in patients with negative cytology and no mass on cross-sectional imaging.

## INTRODUCTION

Cholangiocarcinoma (CCA) is the most common malignancy of the biliary system and extrahepatic CCA presents clinically as biliary strictures [1, 2]. In 50% of cases, CCA involves the confluence of the right and left hepatic ducts (perihilar carcinomas), while the remainder arises from the intrahepatic ducts (8%) or more distally (42%) [3]. These strictures are difficult to diagnose because these patients present with indeterminate strictures that often have no mass on cross-sectional imaging. Also, cytological brushing and/or endoscopic biopsies are often non-diagnostic. Primary sclerosing cholangitis (PSC) patients are at increased risk of developing CCA and they represent a unusual subset of patients [4, 5]. CCA is often unresectable because of delay in diagnosis and is hence associated with a poor prognosis [4, 5].

Brush cytology, obtained during endoscopic retrograde cholangiopancreatography (ERCP), has a low sensitivity for diagnosis of CCA [6]. Fluorescence *in situ* hybridization (FISH) assesses the presence of chromosomal aneuploidy. Even with improvements in cytological techniques, the sensitivity of FISH polysomy for CCA is still low, limiting its usefulness in diagnosis of CCA [7].

Endoscopic ultrasound (EUS) has become a valuable tool in the evaluation of the pancreaticobiliary system. Multiple studies have reported on the use of EUS-fine needle aspiration (FNA) for the diagnosis of extrahepatic CCA [8]. To the best of our knowledge, the literature lacks a systematic review and meta-analysis of the available evidence that has examined the diagnostic performance of EUS in the diagnosis of CCA as the etiology of biliary strictures. The primary aim of our study was to assess the overall diagnostic utility of EUS for biliary strictures. Our secondary aim was to study the role of EUS-FNA in patients in whom the results of brush cytology are negative.

## METHODS

### Literature search

A comprehensive search of the literature was performed to identify articles that examined the diagnostic accuracy of EUS to detect CCA. We systematically searched the PUBMED and EMBASE databases for studies published from January 1980 to April 2014 by using the following search terms “primary sclerosing cholangitis”, “cholangiocarcinoma”, “endoscopic ultrasound” and “fine needle aspiration”. We searched for additional references by cross-checking the bibliographies of retrieved full-text papers. Two reviewers (UN and BN) independently screened the titles and abstracts of all the articles according to pre-defined inclusion and exclusion criteria. Any differences were resolved by mutual agreement and in consultation with the third reviewer (MS).

### Study selection criteria

Studies investigating the use of EUS for detection of CCA or indeterminate biliary strictures were included. The data needed to be sufficient to calculate the sensitivity and specificity. Diagnosis of CCA is challenging because the tumors are less cellular [3]. Only studies that accepted only a ‘positive for malignancy’ cytological interpretation as indicative of malignancy were included and patients who were included if under suspicion for malignancy were excluded. The exclusion criteria were (i) studiea with insufficient data; (ii) reviews, editorials, correspondence letters that did not report their own data and (iii) case reports and studies with fewer than 10 patients.

The index test was use of EUS-FNA with studies reporting ‘positive for malignancy' in our analysis. Confirmation of CCA by histopathology at the time of surgery or inoperable at the time of surgery or autopsy was used as the reference standard.

### Assessment of methodological quality

Two authors (UN and BN) independently assessed study quality using the Quality Assessment of Diagnostic Accuracy Studies (QUADAS-2) assessment tool [4, 5]. Any differences were resolved by a third author (VL). We considered studies that were classified as ‘low risk of bias' and ‘low concern' in all the domains as studies with high methodological quality.

### Statistical analysis and data synthesis

The included studies were analysed according to the methodology suggested by the Cochrane DTA Working Group [10, 11]. This methodology gives more clinically useful results, as it is focused on two statistical measures of diagnostic accuracy: the sensitivity of the test (the proportion of those with the disease who have an abnormal test result) and the specificity of the test (the proportion of those without the disease who have a normal test result).

Only studies in which we were able to obtain data to populate 2x2 tables were included. Initial analysis was performed using the Review Manager (Rev Man 5.2, Copenhagen: The Nordic Cochrane Centre, The Cochrane Collaboration). After preparing and exporting data from Rev Man, we used the NLMIXED procedure in SAS version 9.2 (SAS Institute Inc., Cary, North Carolina, USA) for meta-analysis of diagnostic accuracy studies, to compute the pooled sensitivity and specificity, and to plot the summary receiver operating characteristics curve with summary point and corresponding 95% confidence region. Positive histopathology of a FNA biopsy taken during diagnostic EUS confirms the presence of cancer (true positive). Thus, the index test and the reference standard are one and the same with positive histopathology after EUS-FNA. As a result, false positives are not possible and there is no sampling error associated with specificity because it is, by definition, equal to 1. This was evident in our inclusion studies, which all had specificity of 100%. Therefore we only performed meta-analysis of sensitivities by removing the logit specificity and correlation parameters from the standard bivariate model, thus simplifying the model to a univariate random-effects logistic regression form. The negative likelihood ratio was derived from the model by using the estimated summary sensitivity and assuming a specificity of 1 (-LR = 1-sensitivity/specificity). Large differences between studies are commonly noted in DTA meta-analyses, so heterogeneity is presumed to exist and random effects models are fitted by default [11].

Subgroup analyses were carried out to assess the utility of EUS-FNA for diagnosis of CCA in patients based on the location of strictures, in patients in whom ERCP brushings are non-diagnostic and in whom EUS revealed a mass lesion.

A sensitivity analysis was subsequently carried out to determine whether any single study included in the meta-analysis had significant influence in the analysis. Every study was removed systematically and its impact on the pooled results for the remaining studies was determined to see if there was any significant change in test performance.

## RESULTS

### Eligible studies and quality assessment

**Figure 1.**
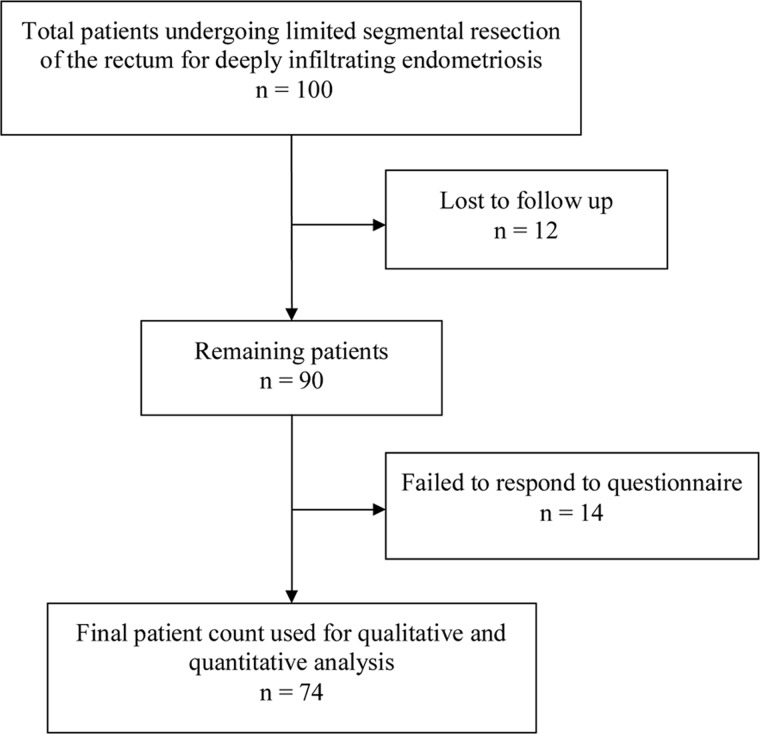
Figure chart of selected studies.

An initial literature search retrieved 1134 articles. We excluded a total of 383 duplicates and clearly irrelevant references by reading the abstracts. We identified 66 references for further assessment. No references were identified by scanning reference lists of the identified studies. Of the 66 references, we excluded 54 for the reasons specified on Figure 1. This resulted in inclusion of 12 studies in the systematic review [12–23]. A previous American Society of Gastroenterology Standards of Practice committee paper had discussed the various studies on the value of EUS in the evaluation of patients with biliary neoplasia [24]. Finally, six studies, listed in Table 1, which dealt only with studies with ‘positive for cancer', were included in the systematic review and meta-analysis.

**Table 1.** Characteristics of included studies in the meta-analysis

Author	Patients with biliary strictures (*n*)	Primary sclerosing cholangitis (*n*)	Mass seen on endoscopic ultrasound (%)	Sensitivity (%)	Specificity (%)
Fritscher-Ravens *et al.*, 2000	10	NR	100	78	100
Fritscher-Ravens *et al.*, 2004	44	4/44	98	83	100
Eloubeidi *et al.*, 2004	28	1/28	89	81	100
Lee *et al.*, 2004	40	3/40	25	29	100
Rösch *et al.*, 2004	50	NR	NR	27	100
Dewitt *et al.*, 2006	24	NR	96	77	100

NR = not reported.

**Table 2.** Characteristics of endoscopic ultrasound-guided fine-needle aspiration in the included studies

Author	On-site cytopathologist present	No. of passes	Cytological interpretations indicative of a positive FNA test result included in the analysis
Fritscher-Ravens *et al.*, 2000	No	1	Only positive
Fritscher-Ravens *et al.*, 2004	No	2–3	Only positive (Both positive and suspicious reported by authors)
Eloubeidi *et al.*, 2004	Yes	≥5	Only positive
Lee *et al.*, 2004	Yes	≥5^a^	Only positive (both positive and suspicious reported by authors)
Rösch *et al.*, 2004	No	≥2^a^	Only positive
Dewitt *et al.*, 2006	Yes	≥1	Only positive

^a^FNA was performed until adequate cellularity was achieved.

**Figure 2.**
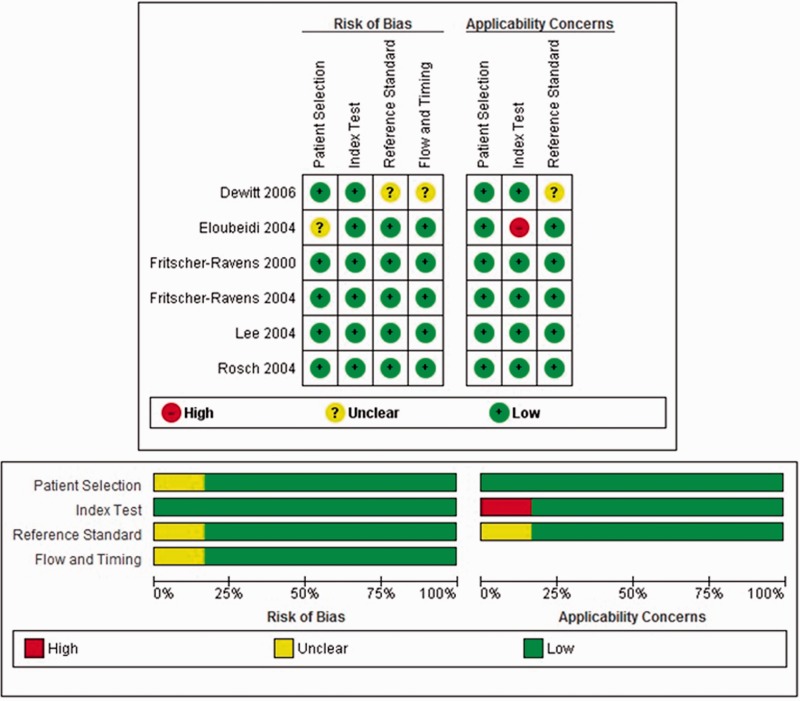
The quality of the eligible studies as assessed by QUADAS-2 criteria.

The methodological quality of the included studies, as assessed by the QUADAS-2 criteria, is shown in Figure 2. In most studies, there was a low risk of bias regarding the selection of patients and we had included only patients who were positive for cancer. There were no bias issues or concerns regarding validity of the selection of patients. There was no bias in any of the studies. Only three studies reported the existence of underlying PSC in their evaluation of CCA [13, 14, 16]. However, the total number of patients included were only 8 in all 3 studies and the studies have not reported their yield separately in this very small sub-group of patients. Hence, for practical purposes our analysis cannot be applicable to PSC patients.

### Sensitivity and negative likelihood ratio

**Figure 3.**
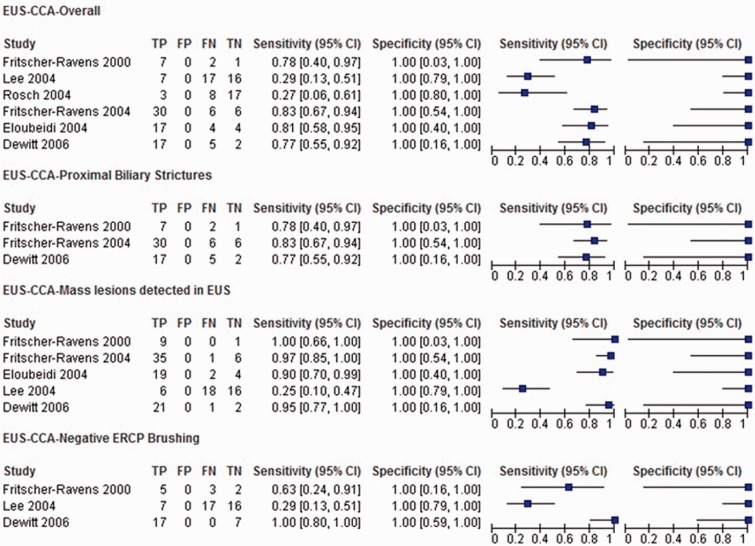
Forest plot of studies reporting the diagnostic role of EUS-FNA; overall in biliary strictures, in proximal biliary strictures, in those with mass lesion seen on EUS and those with a negative bile duct brushings.

**Figure 4.**
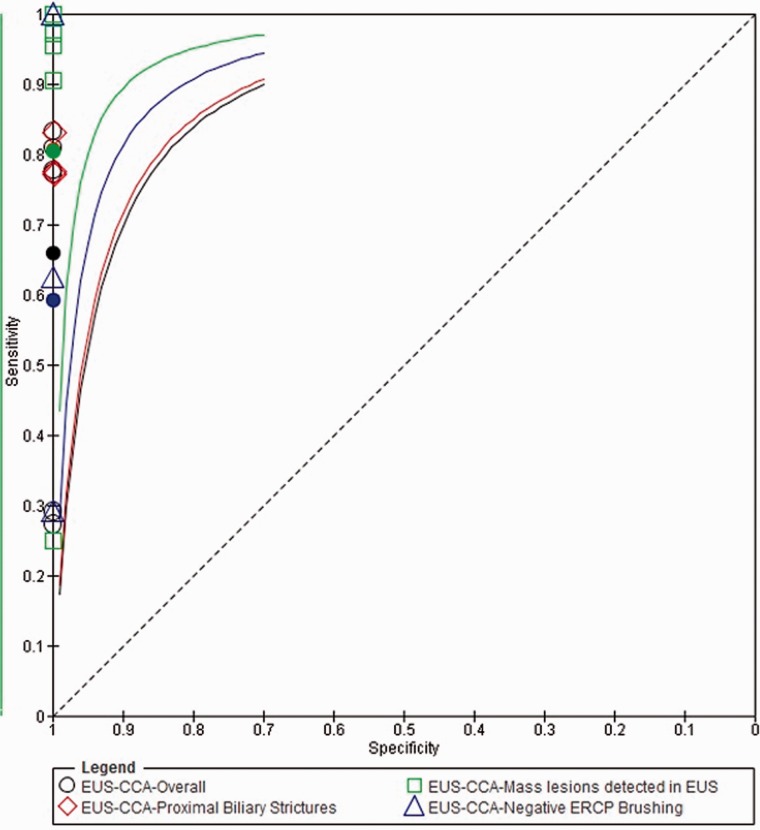
Summary receiver operating curve (SROC) for EUS-FNA to diagnose cholangiocarcinoma.

The overall pooled sensitivity and negative likelihood ratio (LR-) of EUS-FNA for diagnosis of CCA were 66% [95% CI 57–74%] and 0.34 (95% CI 0.26–0.43), respectively (Figures 3 and 4). In our subgroup analysis, limited to studies with a proximal biliary location of the stricture, the pooled sensitivity and negative likelihood ratio (LR-) of EUS-FNA for diagnosis of CCA were 81% [95% CI 69–89%] and 0.19 (95% CI 0.11–0.31), respectively (Figures 3 and 4) [12, 13, 19]. In our subgroup analysis limited to studies with a mass lesion detected during EUS, the pooled sensitivity and negative likelihood ratio (LR-) of EUS-FNA for diagnosis of CCA were 80% [95% CI 72–87%] and 0.20 (95% CI 0.13–0.28), respectively (Figures 3 and 4) [12–14, 16, 19].

For studies with a negative ERCP brush cytology, the pooled sensitivity and negative likelihood ratio (LR-) of EUS-FNA for diagnosis of CCA were 59% [95% CI 44–73%] and 0.41 (95% CI 0.27–0.56), respectively (Figures 3 and 4) [12, 16, 19].

### Sensitivity and specificity in patients without any mass on cross-sectional imaging

Only two studies reported the value of EUS in patients without a mass lesion detected during cross-sectional imaging, the pooled sensitivity of EUS-FNA for diagnosis of CCA was 45% [16, 19].

### EUS-FNA of lymph nodes

Only four studies have reported EUS-FNA of lymph nodes in their analysis [12, 13, 16, 19]. However, only two of these reported the yield of EUS-FNA [12, 19]. The first study reported this in one patient who underwent EUS-FNA of celiac and para-aortic lymph nodes, which were positive for CCA. The other study reported this in one patient, who had EUS-FNA of gastrohepatic lymph nodes, which were positive for CCA. The other two studies reported EUS-FNA of lymph nodes in 12 patients and the yield of EUS-FNA was not reported separately [13, 16].

### Sensitivity analysis

We systematically removed one data set at a time and recalculated the pooled sensitivity and negative likelihood ratio. The largest change occurred when removing the data set from Lee *et al.* [16], which changed the pooled sensitivity from 66% to 75% (+9%), and the corresponding change in -LR value was from 0.34 to 0.25. The second largest change occurred when removing the data set from Rosch *et al.* [17], which changed the pooled sensitivity from 66% to 70% (+4%), and the corresponding change in -LR value was from 0.34 to 0.30. These results indicated that no single data set carried enough weight to significantly influence the pooled test performance reported for EUS-FNA in the diagnosis of CCA.

## DISCUSSION

Tissue-proven diagnosis of extrahepatic CCA is especially difficult. It has been suggested that EUS with FNA improves the diagnosis of CCA as the etiology of extrahepatic biliary strictures. We demonstrated that the pooled sensitivity and specificity of EUS-FNA to detect CCA as the etiology of biliary strictures were 66% and 100%, respectively. Thus the use of EUS improves the diagnostic armamentarium in patients with suspected CCA and is sensitive and very specific in diagnosing CCA.

Bile duct brushings are the most commonly used method for tissue sampling during ERCP and the use of cytology and FISH have a low sensitivity [5–8]. Thus a significant number of patients remain non-diagnostic after these investigations. We observed that, in patients with negative brush cytology, the pooled sensitivity and specificity of EUS-FNA for diagnosis of CCA were 59% and 100%, respectively, highlighting the significant role EUS-FNA plays in diagnosis of CCA, which is challenging because the tumors are less cellular. Also, we only included studies which accepted ‘positive for malignancy’ cytologic interpretation as indicative of malignancy.

The negative likelihood ratio is a measure of how well the same test performs in excluding the disease state. In our study, the negative likelihood ratio (LR-) was 0.34 (95% CI 0.26–0.43). This suggests that, although EUS is highly specific, it cannot be used as a stand-alone test for excluding CCA.

We also observed that the pooled sensitivity and specificity of EUS-FNA for diagnosis of CCA with proximal biliary strictures (above the confluence of the cystic duct and the bile duct) were 81% and 100%. We could not separately study the yield in distal strictures, as most studies which reported on the yield of EUS-FNA in proximal strictures [12, 16, 19]. Experts suggest that the yield in proximal strictures may be lower because the examination is performed from the duodenal bulb with a counterclockwise torque in the endoscope and the lesion is closer to the liver, making sampling technically difficult. However, we still observed a high yield in proximal strictures.

EUS is particularly useful in patients without a definite mass lesion seen on cross-sectional imaging as a definite mass is seen on radiological imaging in only a third of patients with extrahepatic CCA. Since extrahepatic CCAs are periductal cancers with less cellularity, and do not demonstrate mass lesions on cross-sectional imaging studies [3], the role of EUS becomes even more important as we observed that even in studies where cross-sectional imaging did not reveal any mass, EUS was able to identify CCA with a 45% sensitivity.

Even though EUS-FNA is useful in CCA, there have also been concerns over the risk of tumor seeding or needle track seeding. In a study from the Mayo Clinic, of 191 patients with locally unresectable hilar disease, who underwent liver transplant evaluation, 16 underwent biopsy of the hilar CCA (13 percutaneous and 3 by EUS), of which 6 were positive for malignancy [25]. The incidence of peritoneal metastasis was 8% in those who did not undergo biopsy, against 83% in those with a diagnostic transperitoneal FNA (*P* = 0.009) [25]. Based on this literature, the Mayo Clinic transplantation protocol excludes patients who have undergone biopsy of the primary tumor for neoadjuvant therapy and liver transplantation. The concern is that the EUS needle traverses the peritoneum and omental fat that will not be resected at the time of liver transplantation. There are, thus far, no reports on tumor seeding in those with extrahepatic CCA arising distal to the cystic duct insertion. Also, since treatment of the distal tumors involves Whipple’s resection and the duodenum is resected, there is less of a problem in these cases. For proximal tumors, if the patient is a candidate for transplantation protocol, given the risk of tumor seeding, FNA should not be performed until more studies are available.

We have systematically studied the role of EUS-FNA in approaching patients with biliary strictures. Even if EUS-FNA of the primary tumor cannot be performed, EUS also provides the opportunity to evaluate regional lymph nodes [26]. In a study exploring the utility of EUS-FNA for nodal staging in locally unresectable hilar CCA considered for liver transplantation, EUS identified regional lymph nodes in all patients [27]. In a study with unresectable CCA, FNA of lymph nodes identified metastases in 8 patients (17%) which excluded these patients from liver transplantation.

We also evaluated the role of EUS-FNA of lymph nodes in our meta-analysis, particularly in patients in whom EUS-FNA of the primary hilar CCA is not possible. EUS-FNA of lymph nodes provides information on resectability and prevents the use of unwarranted chemoradiation and brachytherapy in patients with lymph node metastasis. Only limited information was available in terms of studies reporting the yield of EUS-FNA of lymph nodes, limiting our interpretation of its clinical implications. However, the studies which reported FNA of lymph nodes in the presence of the mass detected CCA in all patients.

From a clinical point of view, given a pooled sensitivity of 66%, for every 100 people evaluated with EUS who truly have CCA, EUS-FNA will miss 34 people with CCA as they will be wrongly classified as negative for CCA. However, given the 100% specificity for EUS-FNA, a positive result would guide clinical management involving either curative surgery or liver transplantation. In those patients, where the diagnosis is missed, further invasive work-up is required and may involve laparoscopic surgery for diagnostic purposes.

### Limitations of our analysis

The number of passes for diagnosis of CCA in our meta-analysis was very variable, as the included studies covered a long time period, with or without the presence of on-site cytopathology. Also, there is a lack of studies that specifically address the usefulness of EUS in CCA, including information on the location and characteristics of tumors. Most studies included patients with biliary strictures and clarified the role of EUS-FNA in diagnosis. The other issue was that only the positive FNA patients underwent surgery for histological confirmation. So this may lead to ‘differential verification bias’ which needs to be accepted in any studies investigating CCA. The other problem was to adjust for confounders to accurately compare data in the included studies.

To conclude, this meta-analysis summarizes available evidence regarding the diagnostic performance of EUS in the detection of CCA. Our study suggests that EUS-FNA contributes to the diagnosis of CCA in patients with negative cytology and in patients in whom cross-sectional imaging does not reveal any mass lesion.

**Funding:** the study was supported by a research grant to Udayakumar Navaneethan from the American College of Gastroenterology.

**Conflict of interest:** none declared.
